# Expression of genes belonging to the interacting TLR cascades, NADPH-oxidase and mitochondrial oxidative phosphorylation in septic patients

**DOI:** 10.1371/journal.pone.0172024

**Published:** 2017-02-09

**Authors:** Laura A. Nucci, Sidnéia S. Santos, Milena K. C. Brunialti, Narendra Kumar Sharma, Flavia R. Machado, Murillo Assunção, Luciano C. P. de Azevedo, Reinaldo Salomao

**Affiliations:** 1 Escola Paulista de Medicina, Hospital São Paulo, Universidade Federal de Sao Paulo, São Paulo, Brazil; 2 Hospital Israelita Albert Einstein, São Paulo, Brazil; 3 Hospital Sírio Libanês, São Paulo, Brazil; University of Sao Paulo, BRAZIL

## Abstract

**Background and objectives:**

Sepsis is a complex disease that is characterized by activation and inhibition of different cell signaling pathways according to the disease stage. Here, we evaluated genes involved in the TLR signaling pathway, oxidative phosphorylation and oxidative metabolism, aiming to assess their interactions and resulting cell functions and pathways that are disturbed in septic patients.

**Materials and methods:**

Blood samples were obtained from 16 patients with sepsis secondary to community acquired pneumonia at admission (D0), and after 7 days (D7, N = 10) of therapy. Samples were also collected from 8 healthy volunteers who were matched according to age and gender. Gene expression of 84 genes was performed by real-time polymerase chain reactions. Their expression was considered up- or down-regulated when the fold change was greater than 1.5 compared to the healthy volunteers. A p-value of ≤ 0.05 was considered significant.

**Results:**

Twenty-two genes were differently expressed in D0 samples; most of them were down-regulated. When gene expression was analyzed according to the outcomes, higher number of altered genes and a higher intensity in the disturbance was observed in non-survivor than in survivor patients. The canonical pathways altered in D0 samples included interferon and iNOS signaling; the role of JAK1, JAK2 and TYK2 in interferon signaling; mitochondrial dysfunction; and superoxide radical degradation pathways. When analyzed according to outcomes, different pathways were disturbed in surviving and non-surviving patients. Mitochondrial dysfunction, oxidative phosphorylation and superoxide radical degradation pathway were among the most altered in non-surviving patients.

**Conclusion:**

Our data show changes in the expression of genes belonging to the interacting TLR cascades, NADPH-oxidase and oxidative phosphorylation. Importantly, distinct patterns are clearly observed in surviving and non-surviving patients. Interferon signaling, marked by changes in JAK-STAT modulation, had prominent changes in both survivors and non-survivors, whereas the redox imbalance (iNOS signaling, oxidative phosphorylation and superoxide radical degradation) affecting mitochondrial functions was prominent in non-surviving patients.

## Introduction

Sepsis is a complex disease that is characterized by activation and inhibition of different cell signaling pathways according to the disease stage [1]. Recently, a new consensus defined sepsis as a life-threatening organ dysfunction that is caused by a dysregulated host response to infection [2].

The pathophysiology of sepsis is complex and involves the interaction of the host with infecting microorganisms. Infection control depends on the host cell recognizing microorganisms, prompting an appropriate immune response. The interaction of cells from the immune system with bacteria and their products occurs through preserved structures of microorganisms known as pathogen-associated molecular patterns (PAMPs), which are recognized by the pattern recognition receptors (PRRs) of innate immune cells. Lipopolysaccharide (LPS), which is present in the outer membrane of gram-negative bacteria, and Toll-like receptor (TLR)-4, which is present on the cell surface, are the best examples of such an interaction [3, 4]. Upon LPS stimulation, transcription factors such as NF-kB are transferred to the nucleus and trigger the transcription of multiple genes, including inflammatory cytokines, ROS and NO.

One of the main sources of ROS in the phagocytes is the NADPH oxidase, an enzyme complex that consists of five subunits assembled upon cellular activation [5]. Under stress conditions, mitochondria also play an important role in cellular oxidative stress and produce high concentrations of ROS. These are related to mitochondrial damage and dysfunction and contribute to the development of organ dysfunction and poor sepsis outcomes [6–8].

TLR signaling, NADPH-oxidase and oxidative phosphorylation are activated and interact during host response to infection. Bacterial sensing, at the cell surface and upon phagocytoses, will trigger TLR pathways, among other PRRs, and promote the assembly of NADPH-oxidase and production of ROS to kill invading microorganisms. In recent years, in addition to its antimicrobial role, NOX2-derived ROS emerged as a critical player in immune modulation [9]. Accordingly, the pretreatment of neutrophils with antioxidants prevented LPS-induced kinase activation, nuclear translocation of NF-kappaB and the production of proinflammatory cytokines [10]. Mitochondria oxidative phosphorylation and energy generation are both a target and source of ROS. Increased levels of nitric oxide (NO) and superoxide (O_2_^-^) have been reported in septic patients, and their levels are related to outcomes [11, 12]. Oxidative stress can impair the mitochondrial function in multiple ways. NO can modulate mitochondrial respiration and oxygen consumption through reversible binding and inhibition at complex IV, leading to the accumulation of NADH and increases in ROS production [13]. NO and superoxide (O_2_^-^) rapidly react to form peroxynitrite anion (ONOO). Because NO is a relatively stable and highly diffusible free radical and O2^-^ is much shorter lived and has restricted diffusion across biomembranes, the sites of peroxynitrite formation are assumed to be spatially associated with the O2^-^ sources, such as plasma membrane NAD(P)H oxidases and mitochondrial respiratory complexes [14]. Mitochondrial enzymes are particularly vulnerable to peroxynitrite, leading to cessation of electron transport and ATP formation, mitochondrial swelling, and permeabilization of the outer mitochondrial membrane, thus allowing the efflux of proapoptotic molecules, such as cytochrome c [14].

Besides their interactions we also considered that genes modulating different functions may be differentially regulated in septic patients and that at the cellular level, different functions might be differentially regulated. We demonstrated in previous studies that monocytes from septic patients have decreased production of inflammatory cytokines, a preserved phagocytic capacity and an increase in production of both NO and ROS, indicating a modulation in the function of these cells during sepsis [15].

Here, we evaluated genes involved in the TLR signaling pathway, oxidative phosphorylation and oxidative metabolism, aiming to assess their interactions and resulting cell functions and pathways that are disturbed in septic patients. To partially avoid sepsis heterogeneity, we selected patients with sepsis secondary to community-acquired pneumonia (CAP). Gene expression and interactions were analyzed at admission and in follow-up samples for patients surviving or dying from sepsis.

## Materials and methods

### Patients and healthy volunteers

Patients who were admitted from January 2008 to December 2009 to the intensive care units of the Sao Paulo, Albert Einstein, and Sirio-Libanes Hospitals (all in Sao Paulo, Brazil) with a clinical diagnosis of sepsis according to the ACCP/SCCM consensus conference [16] were enrolled in a prospective cohort of septic patients [17, 18]. The study was approved by the ethics committees of the participating hospitals, Sao Paulo Hospital (Study number 1477/06), Albert Einstein Hospital (Study number 07/549) and Sirio Libanes Hospital (Study number 2006/27). Blood samples were collected in sodium heparin-treated tubes (BD Biosciences, Franklin Lakes, NJ, USA), after written informed consent was collected from the patient or representative next of kin. Blood samples obtained from 16 patients with sepsis secondary to CAP were selected for this study. Samples were obtained at admission (D0), and 10 of the patients had a second sample collected after 7 days (D7) of therapy. Samples were also collected from 8 healthy volunteers who were matched according to age and gender.

### Peripheral blood mononuclear cells

Peripheral blood mononuclear cells were obtained using the Ficoll gradient method (Ficoll-Paque PLUS; GE Healthcare Bio-Sciences AB, Uppsala, Sweden), frozen in fetal bovine serum (Invitrogen-Gibco, Gaithersburg, MD, USA) with 10% dimethyl sulfoxide (Calbiochem, La Jolla, CA, USA) and stored in liquid nitrogen prior to use. Upon thawing, the cells were washed and suspended in R10 (RPMI 1640 medium supplemented with 10% fetal bovine serum, 1% HEPES buffer solution 100 mM, 1% L-glutamine 200 mM, 1% sodium pyruvate 100 mM, 1% penicillin/streptomycin 100X, and 0.1% 2-mercaptoethanol 55 mM, all from Invitrogen). The standard cell concentration was 1X10^7^ cells/mL.

### Quantitative RT-PCR array

Total RNA was isolated using silica membrane from an *RNEasy Mini-Kit* (Qiagen, Hilden, Germany). Samples were treated with DNase I (Qiagen). Total RNA quantification was obtained using a Nanodrop device. Purity and integrity were confirmed by spectrophotometry (A260/A280 ratio), and 500 ng of RNA per sample was used for cDNA synthesis. Reverse transcription reactions were performed using an RT^2^ First Strand Kit (Qiagen) according to the manufacturer’s protocol. Sample quality was verified using RT^2^ RNA QC PCR Array plates (Qiagen), which contain controls for RNA integrity, the presence of reverse transcription and PCR amplification inhibitors, and the presence of genomic or other DNA contamination.

Real-time polymerase chain reactions were performed using the RT^2^ Customized Profiler^™^ PCR Array from Qiagen. For each sample, a total of 84 genes were analyzed (listed in Table 1). For customization we choose the genes of interest, while the sequences of primers were the standards of the manufacturer. Amplification, data acquisition, and melting curve analysis were performed in a 7500 real time PCR system (Termo Scientific), and in each run, every gene was checked for the efficiency, threshold point, baseline point and melting curve. To enable a comparison between runs, the same threshold was established for all genes and runs. Two genes (GAPDH and HPRT1) were used as internal controls, and the average of their Ct values was used to normalize gene expression (2^−ΔCt^) and determine the fold change between groups (2^−ΔΔCt^). Gene expression was considered up- or down-regulated when the fold change was greater than 1.5.

**Table 1:** Genes evaluated in the PCR-array, grouped according to their function.

**TLR signaling pathway (22)**						
TLR4	CD14	LY96	MYD88	IRAK3	NFKBIA	NFKB1
TRAF6	ECSIT	IRF5	CHUK	IKBKG	MAPK14	MAP2K7
RELA	MAPK7	MAP2K3	MAPK8	JUN	TICAM1	IRF7
IRF3						
**Interferons signaling pathway (12)**						
IFNAR1	IFNAR2	JAK1	TYK2	STAT1	STAT2	IRF9
IFNGR1	IFNGR2	JAK2	SOCS3	IRF1		
**Oxidative stress (17)**						
NOS1	NOS2	NOS3	SOD1	SOD2	SOD3	CYBB
RAC1	UCP2	GPX1	PRDX3	PRDX4	CAT	TXN
TXNRD1	TXNRD2	NCF1				
**Oxidative phosphorylation (25)**						
NDUFA4	NDUFA5	NDUFA7	NDUFA8	NDUFA9	NDUFA10	NDUFA11
NDUFB5	NDUFB6	NDUFC2	COX4I2	COX5A	COX5B	COX6A2
COX7A2	COX7C	COX10	ATP5B	ATP5C1	ATP5F1	ATP5J
ATP6V1C1	ATP6V1E1	ATP6V1E2	UQCRC2			
**Cytokines (8)**						
TNF	IL1B	IFNA1	IFNB1	IL10	IL6	CXCL10
IL12A						

### Statistical analysis

Differences in gene expression were evaluated using Student’s t test for independent samples with *online software* (Qiagen). A p-value of ≤ 0.05 was considered significant. The list of genes and FC values was imported in the *Ingenuity Pathway Analysis* (IPA) (Ingenuity Systems, Redwood City, CA, USA) for evaluating the signaling cascade and predicting the likelihood of an association between the set of altered genes and related pathways. The Fisher's exact test from IPA was used for this purpose.

## Results

### Casuistic and clinical data

Sixteen patients (D0) with sepsis secondary to pneumonia were enrolled in the study; 12.5% had severe sepsis, and 87.5% had septic shock. Their mean age was 71.7 years, ranging from 45 to 93 years, and 75% were male. The mean SOFA (Sequential (sepsis) Organ Failure Assessment) on day zero was 8±1.9, and the hospital mortality rate was 55.0%. The mean age for healthy volunteers was 64.8 years, ranging from 35 to 86 years and 62.5% were male.

### Gene expression at admission and follow-up

**Fig 1:**
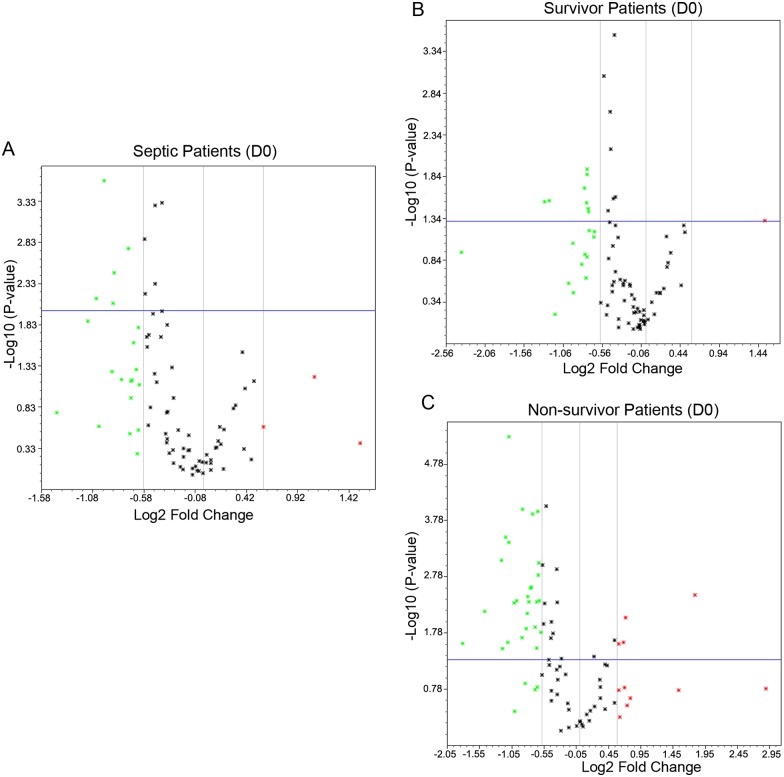
Volcano plot representing the gene expression changes in admission samples (D0) of septic patients compared to healthy volunteers. A. all patients; B. survivors; and C. non-survivors. The x axis represents the Log_2_ fold change and Y axis—Log_10_ *P* value. The cut off for significance is set as the fold change ≥ 1.5 and P value ≤ 0.05. The genes are represented as dots, where the green color represents down regulation, red represents up regulation and gray indicates no significant changes.

Gene expression in septic patients was analyzed in admission (D0) and follow-up samples (D7) and according to patient’s outcomes, survivors and non-survivors. Among the 84 genes that were studied, 22 genes were differently expressed in D0 samples. Among them, 19 were down-regulated, and 3 were up-regulated; 15 of them had p<0.05 (Fig 1A). When gene expression was analyzed according to the outcomes, patients who survived presented with 21 differently expressed genes; only one gene was up-regulated, and nine of them had p<0.05 (Fig 1B). In contrast, 42 genes had differential expression in non-surviving patients, 31 were down-regulated, and 11 were up-regulated; 31 had p<0.05 (Fig 1C).

**Fig 2:**
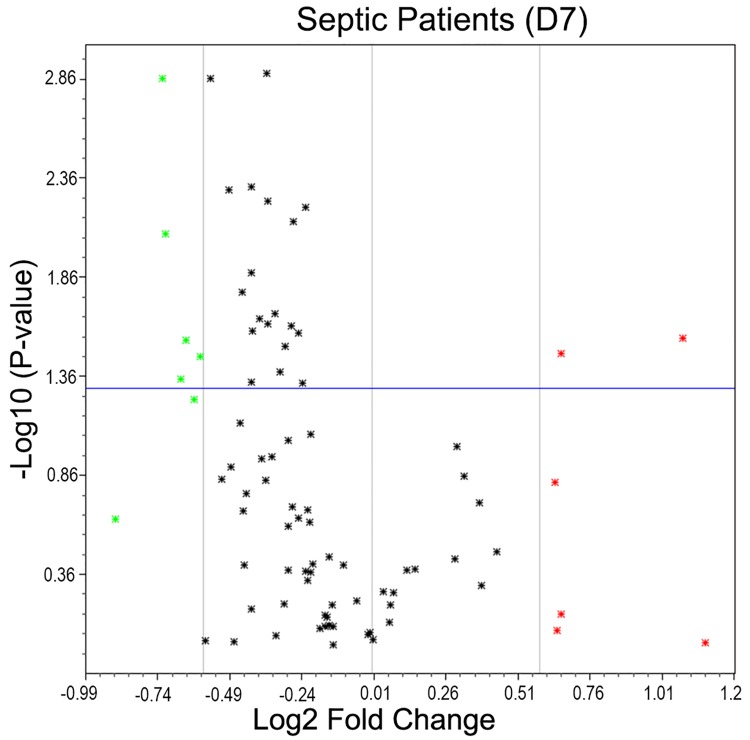
Volcano plot representing gene expression changes in follow-up samples (D7) of septic patients compared to healthy volunteers. A. all patients; B. survivors; and C. non-survivors. The x axis represents Log2 fold change and Y axis—Log10 *P* value. The cut off for significance is set as a fold change ≥ 1.5 and P value ≤ 0.05. The genes are represented as dots, where the green color represents down regulation, red represents up regulation and black indicates no significant changes.

Samples analyzed after 7 days of treatment also showed gene expression modulation compared with the control group. Thirteen genes showed changes; 7 genes were down-regulated, and 6 genes were up-regulated (Fig 2).

### Gene expression and network of interactions in septic patients

**Fig 3:**
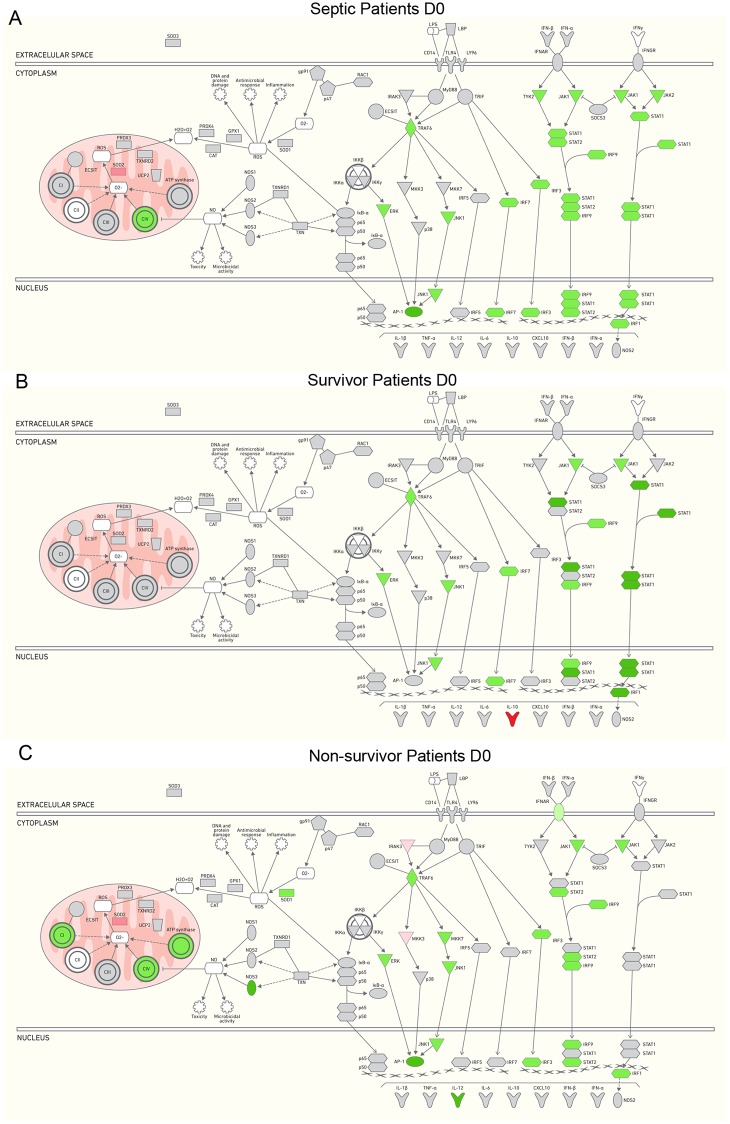
Demonstration of differential gene expression profile in septic patients in admission (D0) samples using curated pathway functional of IPA. A. all patients; B. survivors; and C. non-survivors. Gene expression changes with FC ≥ 1.5 and P value ≤ 0.05 were used to generate the interaction network. The intensity of the color represents up-regulation (red), down-regulation (green) or no significant regulation (gray).

Table 2 shows the fold change ratios in the gene expression of septic patients compared to the healthy volunteers at admission and after seven days of follow-up, and according to the outcomes. IPA was used to represent the altered gene expression involved in signaling pathways for TLR, interferon and oxidative metabolism. At D0, genes related to TLR and the interferon cascade were down-regulated (TRAF6, MAPK7, MAPK8, JUN, IRF7, IRF3 and JAK1, STAT1, STAT2, IRF9, and IRF1), and a few of the genes involved in oxidative phosphorylation were modulated; NDUFC2, COX412 and COX10 were down-regulated, and SOD2 was up-regulated (Table 2, Fig 3A). When gene expression was analyzed according to the outcomes, 9 genes were differently expressed in surviving patients, and all were involved in the cascade of TLR and Interferon. TRAF6, MAPK7, MAPK8, IRF7, JAK1, STAT1, IRF9, and IRF1 were up-regulated and only IL10 was down-regulated (Table 2, Fig 3B). In contrast, 31 genes were modulated in non-surviving patients; they were mostly down-regulated. Down-regulated genes included TRAF6, MAPK7, MAP2K7, MAPK8, JUN, IRF3 and IL12A, which were related to TLR cascade; IFNAR1, JAK1, STAT2, IRF9 and IRF1, related to the interferon cascade; NOS3 and SOD1, related to oxidative metabolism; and NDUFA4, NDUFA5, NDUFA7, NDUFA10, NDUFB5, NDUFB6, NDUFC2, COX7A2, COX7C, COX10, ATP5C1, ATP5F1 and ATP6V1E2, which are specific to mitochondria metabolism. Only 4 genes were up-regulated: IRAK3 and MAP2K3 were related to the TLR cascade, IFNGR2 was related to the interferon cascade and SOD2 was related to mitochondria metabolism (Table 2, Fig 3C).

**Table 2:** Fold change ratios in the gene expression of septic patients compared to the healthy volunteers at admission and after seven days of follow-up, according to the outcomes.

	D0			D7
Gene	All (N = 16)	S (N = 8)	NS(N = 8)	All (N = 10)
TLR4	1.08	-1.06	1.25	1.05
CD14	1.17	1.05	1.34	-1.11
LY96	1.1	1.02	1.24	-1.12
MYD88	1.1	-1.06	1.25	-1.01
IRAK3	1.41	1.21	**1.64** ^**a**^	1.3
TRAF6	**-1.81** ^**a**^	**-1.70** ^**a**^	**-2.14** ^**a**^	-1.47
ECSIT	-1.3	-1.31	-1.35	-1.23
IRF5	-1.22	-1.08	-1.35	-1.11
CHUK	-1.04	-1.21	1.01	-1.1
IKBKG	-1.12	-1.09	-1.14	1.09
NFKBIA	1.22	-1.21	**1.62**	1.35
NFKB1	1.09	1.17	1.08	-1.16
RELA (p65)	-1.41	-1.38	-1.45	-1.34
MAPK7 (ERK)	**-1.86** ^**a**^	**-1.69** ^**a**^	**-2.14** ^**a**^	**-1.66** ^**a**^
MAP2K3 (MKK3)	1.29	1.2	**1.52** ^**a**^	1.05
MAPK14	1.24	1.21	1.32	1.25
MAP2K7 (MKK7)	-1.49	-1.39	**-1.66** ^**a**^	-1.25
MAPK8 (JNK1)	**-1.74** ^**a**^	**-1.69** ^**a**^	**-1.85** ^**a**^	-1.41
JUN (AP1)	**-2.05** ^**a**^	**-1.70**	**-2.78** ^**a**^	-1.43
TICAM1	-1.11	-1.33	1	-1.17
IRF7	**-1.54** ^**a**^	**-1.73** ^**a**^	-1.50	-1.35
IRF3	**-1.61** ^**a**^	**-1.58**	**-1.70** ^**a**^	-1.37
IFNAR1	-1.23	-1.08	**-1.51** ^**a**^	-1.27
IFNAR2	-1.2	-1.12	-1.39	**-1.53**
JAK1	**-1.80** ^**a**^	**-1.68** ^**a**^	**-2.22** ^**a**^	**-1.64**
TYK2	-1.37	-1.31	-1.47	-1.29
STAT1	**-1.78** ^**a**^	**-2.46** ^**a**^	**-1.80**	-1.13
STAT2	**-1.58** ^**a**^	**-1.72**	**-1.77** ^**a**^	-1.36
IRF9	**-1.52** ^**a**^	**-1.66** ^**a**^	**-1.61** ^**a**^	-1.19
IFNGR1 (IFNGR)	1.04	1.13	1.04	1.11
IFNGR2 (AF-1)	1.46	1.4	**1.60** ^**a**^	1.23
JAK2	-1.48	**-1.69**	**-1.57**	-1.17
SOCS3	-1.07	-1.02	-1.22	-1.23
IRF1	**-1.94** ^**a**^	**-2.37** ^**a**^	**-2.17** ^**a**^	-1.36
NOS1	1	-1.35	1.46	-1.4
NOS2	-1.09	-1.02	-1.12	-1.34
NOS3	**-2.54**	**-1.91**	**-3.52** ^**a**^	**-1.85**
SOD1	**-1.6**	-1.4	**-2.02** ^**a**^	**-1.56** ^**a**^
SOD2	**2.15** ^**a**^	1.37	**3.45** ^**a**^	**2.12** ^**a**^
SOD3	-1.35	**-1.59**	1.03	-1.12
CYBB (gp91)	-1.1	-1.35	1.11	-1.1
NCF1 (p47phox)	1.15	-1.06	**1.52**	**1.56**
RAC1	-1.03	-1.05	-1.12	-1.04
UCP2	-1.31	-1.42	-1.38	-1.1
GPX1	1.34	1.41	1.46	**1.58** ^**a**^
PRDX3	-1.07	-1.02	-1.27	-1.17
PRDX4	-1.04	1.13	-1.35	1
CAT	1	-1.02	-1.03	-1.15
TXN	1.11	1.1	1.17	1.03
TXNRD1	-1.12	-1.11	-1.22	-1.07
TXNRD2	-1.22	-1.22	-1.26	-1.21
NDUFA4	-1.41	-1.37	**-1.55** ^**a**^	-1.31
NDUFA5	-1.41	-1.06	**-1.86** ^**a**^	-1.22
NDUFA7	-1.35	-1.34	**-1.54** ^**a**^	-1.26
NDUFA8	-1.18	-1.17	-1.28	-1.11
NDUFA9	-1.38	-1.37	-1.49	-1.21
NDUFA10	-1.39	-1.28	**-1.57** ^**a**^	-1.28
NDUFA11	-1.19	-1.15	-1.16	-1.21
NDUFB5	-1.45	-1.35	**-1.73** ^**a**^	-1.33
NDUFB6	-1.37	-1.31	**-1.58** ^**a**^	-1.22
NDUFC2	**-1.90** ^**a**^	**-1.77**	**-2.31** ^**a**^	**-1.58** ^**a**^
COX4I2	**-1.83** ^**a**^	**-1.91**	**-1.62**	-1.39
COX5A	-1.15	-1.02	-1.36	-1.16
COX5B	-1.29	-1.33	-1.28	-1.19
COX6A2	1.18	-1.01	1.31	-1.26
COX7A2	-1.29	-1.12	**-1.59** ^**a**^	-1.36
COX7C	-1.49	-1.4	**-1.67** ^**a**^	-1.34
COX10	**-1.66** ^**a**^	**-1.65**	**-1.74** ^**a**^	-1.30
ATP5B	-1.2	-1.16	-1.27	-1.17
ATP5C1	-1.32	-1.22	**-1.56** ^**a**^	-1.28
ATP5F1	-1.4	-1.26	**-1.76** ^**a**^	-1.34
ATP5J	-1.18	-1.12	-1.33	-1.18
ATP6V1C1	1.22	1.24	1.12	1.04
ATP6V1E1	-1.17	-1.13	-1.23	-1
ATP6V1E2	**-1.56**	-1.28	**-1.97** ^**a**^	**-1.51** ^**a**^
UQCRC2	-1.41	-1.46	-1.43	-1.29
TNF	1.04	**-1.99**	**1.54**	1.3
IL1B	1.38	**-2.24**	**2.9**	**1.56**
IFNA1	-1.04	-1.05	1.18	-1.16
IFNB1	1.12	-1.49	**1.67**	**1.58**
IL10	**1.96**	**2.87** ^**a**^	**1.73**	1.22
IL6	**3.6**	1.08	**7.42**	**2.23**
CXCL10	**-2.49**	**-5.16**	**-2.02**	-1.49
IL12A	**-1.5**	-1.28	**-2.29** ^**a**^	-1.22

Footnote: D0 represents total septic patients after admission and D7 represents samples obtained after 7 days. S: survivors and NS: non-survivors. The bold letters show fold change ≥1.5 and the letter “a” indicates P value ≤0.05.

**Fig 4:**
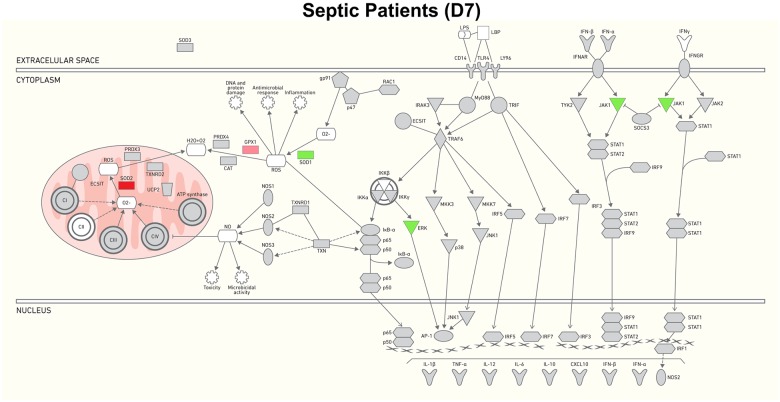
Gene expression changes and network of interactions between control and septic patients after 7 days. Gene expression changes with FC≥ 1.5 and P value ≤ 0.05 were used to generate the interaction network. The intensity of the color represents up-regulation (red), down-regulation (green) or no regulation (gray).

Seven genes were differently expressed in the follow-up samples. Among them, 5 were down-regulated, including MAPK7 and JAK1, related to the TLR/Interferon cascade, and SOD1, NDUFC2 and ATP6V1E2, related to the oxidative/mitochondria metabolism cascade; 2 genes were up-regulated, i.e., SOD2 and GPX1, which are both related to the oxidative/mitochondria metabolism cascade (Fig 4).

### Canonical pathways differently regulated during sepsis

We evaluated the canonical pathways that were affected by changes in the expression of the 84 genes studied in the D0 and D7 samples and according to the patient outcomes, i.e., survivors and non-survivors.

**Fig 5:**
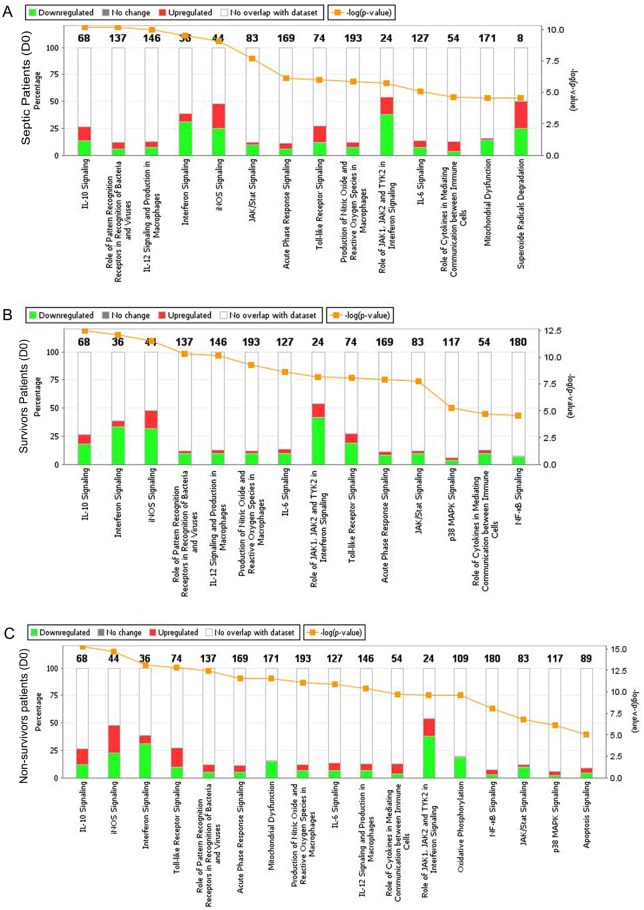
Canonical pathways affected by changes in gene expression during sepsis. A. all patients; B. survivors; and C. non-survivors. The percentage of up-regulated and down-regulated genes in each selected canonical pathway can be measured in the percentage scale given on the y-axis (left side), in which red corresponds to the up-regulated genes and green corresponds to the down-regulated genes. The yellow line represents the −log (p-value) of affected genes in a given pathway. Canonical pathways with −log (p-value) higher than 4.5 are represented.

The canonical pathways altered in D0 samples included IL-10 signaling, interferon and INOS signaling; the role of JAK1, JAK2 and TYK2 in interferon signaling; mitochondrial dysfunction; and superoxide radical degradation pathways (Fig 5A). When analyzed according to outcomes, different pathways were prominent in surviving and non-surviving patients. IOf note, the mitochondrial dysfunction, oxidative phosphorylation and superoxide radical degradation pathway were more altered in non-surviving patients (Fig 5B and 5C).

Four canonical pathways were disturbed at D7, and there was a greater representation of down-regulated genes in superoxide radical degradation; a role of JAK1, JAK2 and TYK2 in the interferon signaling pathway; interferon signaling; and INOS signaling (data not shown).

Direct comparison between survivors and non-survivors showed differences in the canonical pathways (S1A Fig), and lower degree of inflammatory response (quantity of nitric oxide, recruitment and activation of phagocytes) in survivors than in non-survivors (S1B Fig).

## Discussion

Our study evaluated the expression of genes related to TLR signaling, mitochondrial oxidative phosphorylation and oxidative stress in septic patients. We found differences between patients and healthy volunteers and, importantly, between survivors and non-survivors. This is consistent with previously reported transcriptomics studies from our group, which showed that principal component analyses segregated survivors from non-survivors [19], and other studies, including those on trauma and septic patients, in which gene expression was different between complicated and uncomplicated cases or survivors and non-survivors [20, 21]. In a recent study, the global gene expression of blood leukocytes from patients with CAP showed marked changes compared with healthy volunteers. Common and unique altered genes were shown in CAP and no-CAP patients, allowing the identification of biomarkers discriminating CAP from no-CAP patients, without discriminating between CAP survivors and non-survivors [22].

In the present study, we found TLR and interferon cascades were down-regulated in admission samples. Few of the genes involved in oxidative phosphorylation were differentially modulated. Down-regulation of the TLR signaling pathways in the PBMCs of septic patients was seen in a previous study performed by our group [23]. Despite preserved TLR gene expression, key downstream genes such as NF-κB have been consistently reported to be down—regulated. Accordingly, we found preserved TLR2 and TLR4 detection on the monocyte cell surface and impaired response to LPS in septic patients [24].

Overall, the changes in gene expression was qualitatively similar in survivors and non-survivors, with differences appearing in the magnitude of the disturbance, as previously reported by Xiao et al in a transcriptome study of trauma patients [20]. Accordingly, lower number of genes was modulated in surviving than in non-surviving patients (shown in Fig 1 and Table 2), and most genes altered in survivors were also altered in non-survivors. As expected, these changes resulted in similar canonical pathways affected in both groups, yet with different position in the top altered ones (Fig 5).

LPS induces TLR4 signaling through two pathways: the MyD88-dependent pathway induces an early activation of nuclear factor-κB (NF-κB), which leads to the production of inflammatory cytokines, and the MyD88-independent pathway or TRIF-dependent pathway induces the activation of interferon (IFN)-regulatory factor 3 (IRF3) and the late activation of NF-κB, which leads to the production of IFN-beta and IFN-inducible genes [1]. We were interested to evaluate representative genes in IFN-induced cascade because of the controversy regarding TRIF-dependent pathway regulation during LPS tolerance. Depending on experimental approaches, authors have found that the induction of TRIF-dependent IFN-α/β and IFN-induced genes is enhanced in LPS-tolerant cells [25], while others reported that non-tolerant genes are not regulated through IFN-α/β feedback [26]. In a previous work analyzing human tolerant PMBCs, we could see that TRIF and IFN-β were down-regulated upon re-stimulation with LPS [27]. In the present work, we found down-regulation of these genes in all septic patients, and accordingly to the outcomes, in survivors and in non-survivors (Table 2 and Fig 3) compared with the healthy volunteers. Accordingly, interferon signaling and role of JAK1, JAK2 and TYK2 in the interferon signaling pathways are among the top relevant canonical pathways altered in our patients (Fig 5). It is known that the JAK / STAT pathway is related to the release of cytokines and inflammatory mediators, such as IL-6, IL-10 and iNOS. There are few studies current evaluating its role in sepsis [28]. In experimental studies, inhibition of JAK2 with AG490 or STAT3 with rapamycin attenuated organ damage and severe sepsis mortality [29, 30]. Additionally, septic mice deficient in TYK2 and STAT1 had a higher survival rate than did wild-type animals. This improvement in survival was associated with less hypothermia, hypoglycemia and hepatocellular injury [31].

Notably, genes encoding the subunits of the mitochondria complex, which are involved in oxidative phosphorylation, were negatively modulated in non-surviving and much less affected in surviving patients (Table 2, Fig 3). In fact, genes related to mitochondrial oxidative phosphorylation from complexes I, IV and V were down-regulated, as were those involved in scavenging mtROS, such as SOD1 and SOD3, catalase, peroxiredoxin (PRDX)-3 and 4, thioredoxin reductase (TXNDRD) 1 and 2. Accordingly, mitochondrial dysfunction and oxidative phosphorylation components were among the most altered canonical pathways in non-surviving patients.

There are several studies showing impairment in the mitochondrial function of patients dying from sepsis. Brealey et al. observed reduced activity of complex I in the mitochondria and ATP content in the skeletal muscle in non-surviving patients [32]. Studies in PBMCs and monocytes of septic patients showed changes in mitochondrial function, such as a reduction in the respiration and/or enzymatic activity of all complexes and in the potential membrane [33–36]. Experimental work allows for studying mitochondria in other tissues. A reduction in the efficiency of oxidative phosphorylation and activity of complex IV has been shown in the brain tissue of mice [37]. Commin et al. observed that rats that suffered CLP showed decreased activity of complex I, but not of the complexes II, III and IV in the electron transport chains [38]. In contrast, increased consumption of oxygen and breathing efficiency was demonstrated in the livers of septic rats [39].

Expression of p47phox (NCF1) and gp91 (CYBB) was not altered in septic patients in the admission samples, except for the increased expression of NCF1 in non-survivors. This might indicate that ROS generation at the phagolysosomes might be preserved. This is supported by our previous results, showing increased assembly of NADPH-oxidase, preserved phagocytic activity and increased ROS generation in monocytes from septic patients [15].

In conclusion, our data show changes in the expression of genes belonging to the interacting TLR cascades, NADPH-oxidase and oxidative phosphorylation. Importantly, distinct patterns are clearly observed in surviving and non-surviving patients. Interferon signaling, marked by changes in JAK-STAT modulation, had prominent changes both in survivors and non-survivors, whereas oxidative phosphorylation affecting mitochondrial functions was prominent in non-surviving patients.

Our study has limitations. One is the low sample size. In part, it is because we selected only those patients with CAP as the source of infection from the cohort of septic patients, aiming to minimize the patient heterogeneity. Additionally, our results are largely based on gene expression. Nevertheless, the conclusions driven from our present data are in agreement with previous functional studies from our group and others.

## Supporting information

S1 Fig Comparative analysis between survivor and non-survivor patients using IPA. A. altered canonical pathways; B. functional alterations.
(DOCX)
