# Supplementary figures and images for: Expression of genes belonging to the interacting TLR cascades, NADPH-oxidase and mitochondrial oxidative phosphorylation in septic patients
Source: PLoS One. 2017 Feb 9;12(2):e0172024. doi: 10.1371/journal.pone.0172024 (PMC5300193; doi:10.1371/journal.pone.0172024)

S1A Fig.


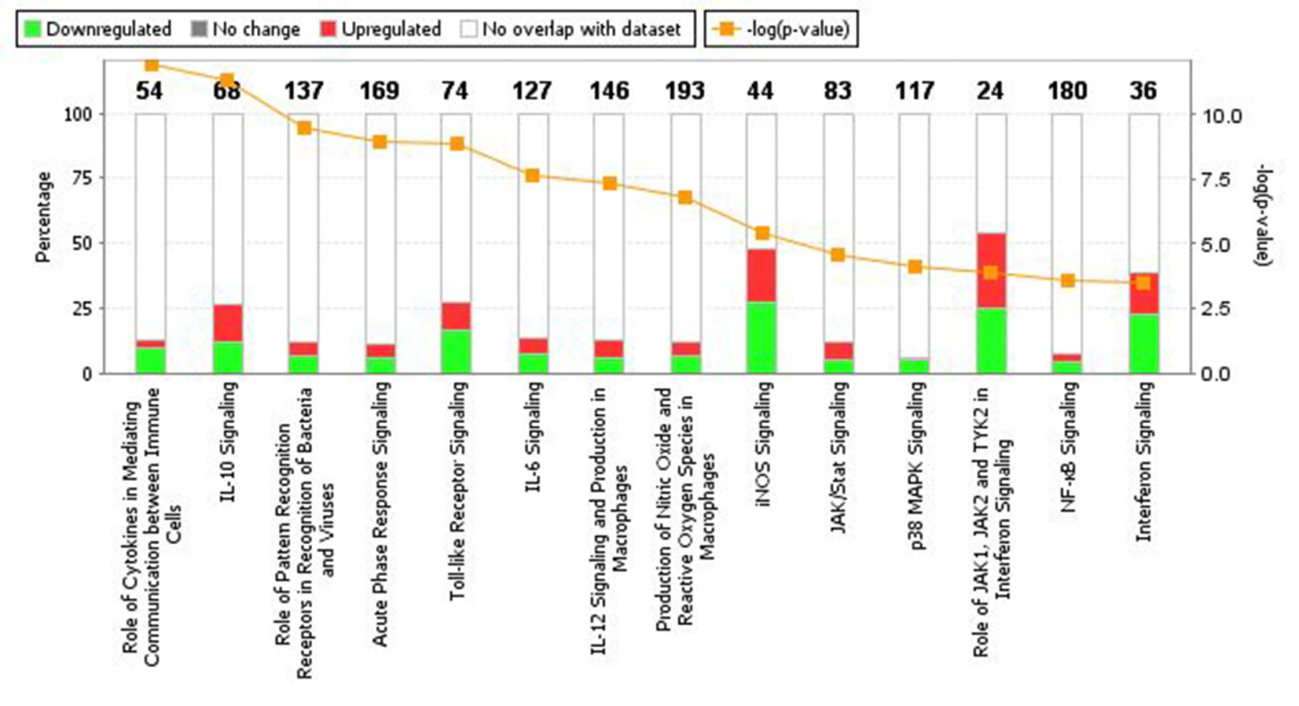


S1B Fig.


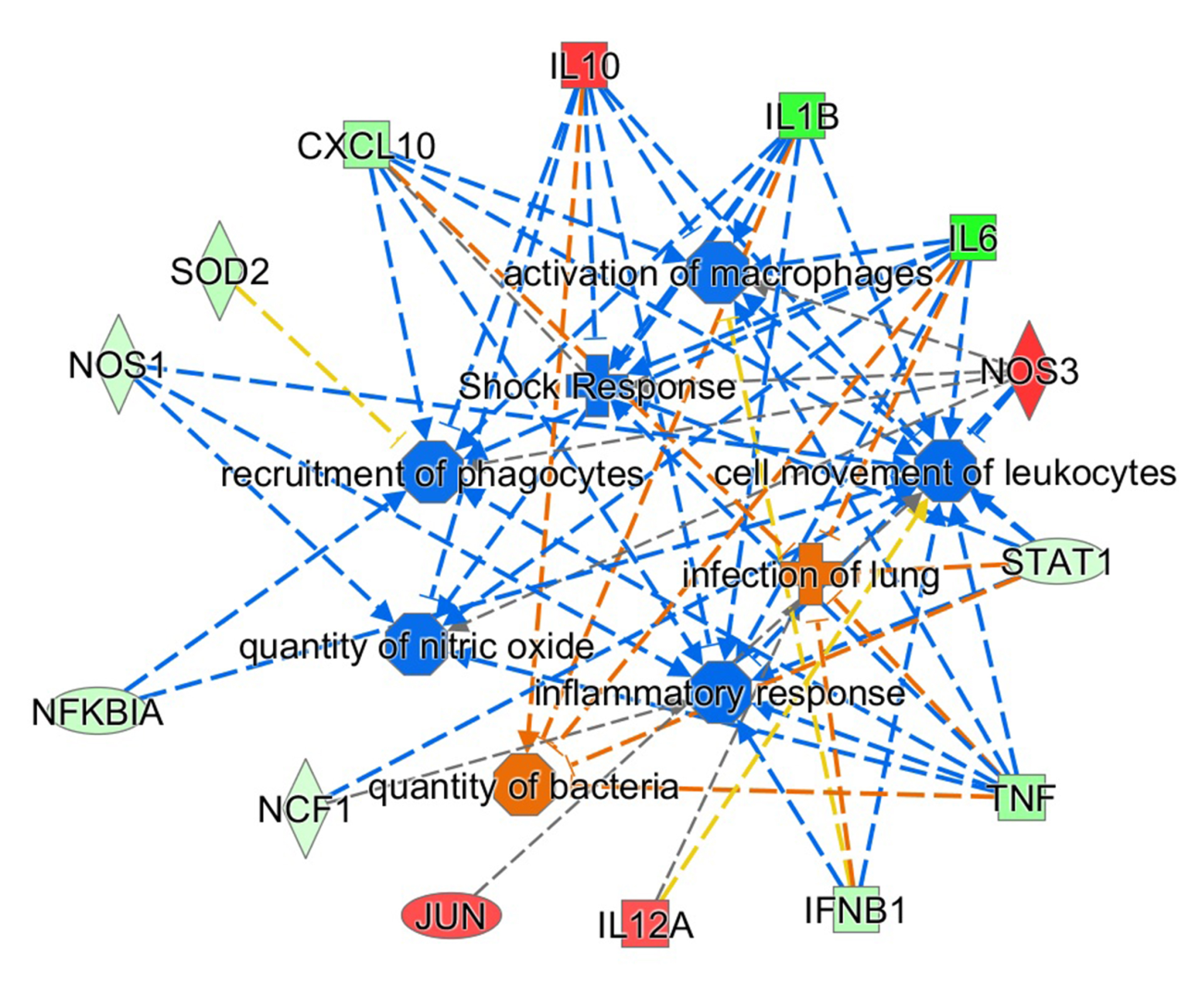

Supplement: S1 Fig — A. altered canonical pathways; B. functional alterations. (DOCX) [file pone.0172024.s001.docx]
